# The Quality of Life of Men Who Have Sex with Men in China: Reliability and Validity Testing of the SF-36 Questionnaire

**DOI:** 10.1371/journal.pone.0083362

**Published:** 2013-12-19

**Authors:** Jie Liu, Bo Qu, Bingxue Hu, Nan Jiang, Dongbo Wang

**Affiliations:** Faculty of Health Statistics, School of Public Health, China Medical University, Shenyang, China; University of Southern California Keck School of Medicine, United States of America

## Abstract

**Objective:**

The aim of the study was to assess the psychometric properties of the 36-Item Short Form Health Survey (SF-36) in the men who have sex with men (MSM) population in China.

**Methods:**

A cross-sectional survey was conducted among 373 MSM from September to December, 2012, in Zhengzhou and Huludao City, China. Internal reliability of the questionnaire was calculated by Cronbach’s α coefficient. Validity was analyzed through construct validity, divisional validity, and collective validity testing.

**Results:**

The overall Cronbach’s α coefficient of the SF-36 questionnaire was 0.943, while the Cronbach’s α coefficients for each of the dimensions were all > 0.70. Results showed that the SF-36 questionnaire was reliable and valid.

**Conclusions:**

This study provided evidence that the SF-36 is an acceptable, valid and reliable instrument in evaluating the quality of life of MSM in Mainland China.

## Introduction

Men who have sex with men (MSM) have not only been impacted upon by HIV epidemics, but the growing prevalence of HIV in MSM shows that they are a driving population for the HIV/AIDS epidemic in the world[1]. MSM are at high risk for HIV infection worldwide because of their high number of partners, unprotected anal intercourse, and high migration rates[2,3]. They also may play a bridging role in the spread of HIV and other sexually transmitted diseases ( STDs ) by transferring diseases from their male sexual partners to their wives[4,5].

Men who have sex with men (MSM) have received increasing attention in China because of a high HIV infection rate. The percentages of newly reported HIV cases attributable to MSM were 0.2% in 2001, 7.3% in 2005, 12.2% in 2007, and 32.5% in 2009 [6]. In 2011, MSM accounted for 29.4 % of the 48,000 newly reported HIV cases and 13% of all reported HIV cases in China[7]. With the population of MSM estimated to be between 5 and 10 million in China, there presents a concentrated public health problem[8]. 

On one hand, the MSM population’s fear of losing social status, feelings of guilt towards family, loneliness, and perceptions of immorality and abnormality all cause MSM to suffer from serious depression, anxiety, stress, low self-esteem, and social isolation as compared to the general population[9]. A study suggested that about 40% of MSM would develop major depression in their lifetimes – twice the rate reported in other men[10]. On the other hand, alcohol misuse, family violence, adverse events such as financial hardship, homelessness, stressful life events extensively exist among MSM population, which contribute to worsened physical conditions[11]. 

Some HIV prevention interventions, such as condom distribution and sexual health education programs, have been somewhat efficacious in increasing HIV knowledge, HIV testing, and condom use and in reducing high-risk sexual activity[12]. However, the efficacies of interventions to reduce incidence of HIV infections were not significant[13,14]. A previous study suggested that coordinated behavioural, biomedical and structural interventions that incorporate efficacious strategies could substantially reduce the incidence of HIV infection in MSM[12]. In China, recent studies have focused much attention on biomedical and behavioural intervention strategies, but few studies have used validated instruments to reflect psychosocial health problems associated with HIV risk behavior among the MSM population[15]. Studies have suggested that psychosocial health problems interact synergistically to increase HIV-related sexual risk behavior among MSM[15,16]. Mental health-related quality of life (HRQL) was found to be independently associated with unsafe or unprotected sex[17]. So, assessing the quality of life of the MSM population can inform us of their health conditions, help us understand the related risk factors, and promote HIV prevention. 

Some studies showed that the MSM population is more concerned about their physical health than the men from the general population, and studies on quality of life (QoL) of MSM population may provide additional knowledge that is of interest in counseling and care[18]. However, most previous studies have focused on QoL of HIV-infected MSM. It is not common to focus on health-related quality of life (HRQL) of HIV zero-status MSM. Even though some studies have indicated that this group’s quality of life is affected, there is limited knowledge about the HRQL of MSM[19]. 

Quality of life (QoL) is defined as either the subjective perception of one’s own well-being within socio-cultural context or as the satisfaction of desires and pleasures and the accomplishment of the ideal to a standard of perfection[20]. As a multidimensional assessment of physical, psychological, and social functions, QoL is believed to be a good measure of studying an individual’s state of health[21]. The SF-36 questionnaire provides a concise method that is mainly used to check the health status of members of the general population, and has become the most widely-used QoL evaluation tool in the world[22,23]. The Chinese version of the SF-36 has been previously administered to the general population and is already widely accepted and proven reliable and valid[24-27]. Now we are testing the applicability of this questionnaire on the specific MSM population in Mainland China. 

In HIV/AIDS studies, the SF-36 displays good internal consistency, reliability, and construct validity[28-31]. Several QoL instruments have been applied in the evaluation of HIV-infected patients, including the multiple versions of the Medical Outcome Study (MOS) [30,32-34], the AIDS-HAQ[35], the HOPES[36], EQ-5D, the SF-36, and the World Health Organization Quality of Life Instrument (WHOQOL) [37]. Each questionnaire has its unique construct and advantages. However, some studies suggested that generic health-related QoL instruments, such as the WHOQOL-BREF and the SF-36, could be quite useful for comparing HIV-infected patients with patients of other diseases and for cost-effectiveness and economic analysis because disease-specific QoL instruments, such as the MOS-HIV did not allow for cross-disease comparison [34]. 

The SF-36 questionnaire can not only provide a direct quantitative indication of MSM’s state of physical health condition through physical functioning, role-physical, bodily pain, general health perceptions scales, but also provide indication of MSM’s state of mental condition through vitality, social functioning, role-emotional, and mental health scales. Therefore, the SF-36 would provide necessary feedback on MSM mental health, which is overlooked by other tools. However, research on literature to assess the psychometric properties of the SF-36 questionnaire on Chinese MSM population in the health professions had rarely been reported. It is important to know the quality of life and promote HIV prevention among Chinese MSM population. 

Thus, in this study, our aim was to test the reliability and validity of the SF-36 questionnaire for the MSM population. With this information, we believe that their physical and mental conditions could be better understood, and better intervention policies could bedeveloped, which should give full consideration on promoting their health.

## Materials and Methods

### Respondents

A cross-sectional study was conducted in Zhengzhou and Huludao City, China, from September to December, 2012. Participants were recruited by trained staff through venue-based recruitment, complemented by internet advertisement, and community outreach. Participants were recruited from gay-oriented venues including clubs, bars, parks, and saunas where MSM meet one another. The respondents underwent a face-to-face explanation prior to taking the standardized questionnaire. Participation in the study was completely voluntary and a written informed consent was obtained from each respondent before the survey. An incentive of 50 Yuan (equivalent to about 8 USD) was given to respondents as compensation for the time spent being interviewed. All interviews were self-administered in a private room in the Center for Disease Prevention and Control (CDC) of Zhengzhou and Huludao City. 

The overall questionnaire included socio-demographic information (age, marital status, nationality, and education). The SF-36 portion of the questionnaire included 36 questions related to an individual’s QoL and contained eight scales: physical functioning (PF), role-physical (RP), bodily pain (BP), general health perceptions(GH), vitality (VT), social functioning (SF), role-emotional (RE), and mental health (MH). 

The raw scores for each scale are transformed to a scale of 0-100, with higher scores indicating ‘better’ QoL. It is summarized in two component summary scores, the Physical Component Summary (PCS) and the Mental Component Summary (MCS).

After the respondents completed the questionnaires, specially trained personnel inspected the questionnaires, identified the questionnaires that were filled out with non-standard or ambiguous answers, and contacted the appropriate investigators and respondents for timely verification. The study protocol was approved by the bioethics advisory commission of China Medical University.

### Statistical Analysis

Validity was analyzed through collective validity (the correlation of each item with its hypothesized scale, corrected for overlap), divisional validity (the evaluation of how distinct each scale was from other scales), and construct validity (the extent to which the questionnaire supports predefined hypotheses)[38]. After deducting the overlap between each of the 36 items and its related scales, the collective validity was considered to be good if the correlation coefficient remains >0.4. To support the divisional validity, items should have higher correlation with their hypothesized scales than with scales measuring other concepts. The statistical significance of the difference between the item-hypothesized scale and item-competing scale correlations was tested by the Steiger’s t-test for dependent correlation[39]. Exploratory factor analysis was the statistical method used to test construct validity. The Kaiser-Meyer-Olkin-Kriterium (KMO) statistic and Bartlett’s spherical check were carried out to check for sample suitability for the factor analysis. 

Internal reliability of the SF-36 questionnaire was measured by determining internal uniformity, which is expressed by Cronbach’s α coefficient. Cronbach’s α coefficient was calculated for the eight scales of the SF-36 questionnaire, and the reliability was considered to be adequate if the α value was >0.7[40]. Split-half reliability, a measure of consistency where a test is split in two and the scores for each half of the test compared with one another, was used to check the internal stability of the questionnaire, and test-retest reliability was used to assess the consistency of the questionnaire from one time to another[41]. In order to determine test-retest reliability, a second round of evaluations was undertaken among 40 (10%) study subjects who were randomly selected 2 weeks later. The data was analyzed using SPSS® version 16.0 (SPSS Inc., Chicago, IL, USA) for Windows®. A *P*-value of < 0.05 was considered to be statistically significant.

## Results

### Socioeconomic characteristics

A total of 379 MSM were surveyed for the study. In total, 373 MSM completed the questionnaire (response rate: 98.4%). The ages of respondents ranged from 18 to 73, with a mean age of 28.2 years. 119 (31.9%) of respondents were local residents of the study sites, and 254 (68.1%) lived in a different province. 155 (41.6%) of the respondents self-identified as bisexual. 272 (72.9%) respondents were single, 77 (20.6%) were married, and 24 (6.4%) were divorced or widowed. The education levels of the respondents were as follows: elementary (19.8%), high school (56.6%), and college or postgraduate (23.6%). Monthly income levels were categorized as: below 2000 Yuan (29.2%), 2000 to 3000 Yuan (42.9%), and 3000 Yuan and above (27.9%). Respondent occupations were as follows: factory workers (20.4%), farmers (7.5%), administrators (11.3%), service personnel (35.9%), teachers, and students (24.9%). 

### Validity Analysis

Construct validity was evaluated by means of factor analysis according to the degree of similarity between the hypothetical structure of the questionnaire conceived by researchers and the actual observed data. Results showed the KMO measure to be 0.877 and the Bartlett’s spherical check to be χ^2^ = 2026.265 and P = 0.000, which, when taken together, indicated that the samples in this study were suitable for factor analysis. Factor analysis results indicated that when two component summary scores, the PCS and the MCS, were extracted from those of the eight scales whose characteristic roots were > 1 or approaching 1, the accumulative contribution rate was up to 72.586%. The PCS had larger factor loads on PF (0.736), RP (0.854) and BP (0.891) scales, with high correlations in accordance with the theoretical hypothesis, and lower factor loads on RE (0.247) and MH (0.233) scales, with low correlations in accordance with the theoretical hypothesis. The MCS scales had larger factor loads on MH (0.887) and RE (0.800) scales, with high correlations in accordance with the theoretical hypothesis, and lower factor loads on RP (0.208), BP (0.247), and PF (0.273) scales, with low correlations in accordance with the theoretical hypothesis. The correlation coefficient (r > 0.50) for each item and its related scale was obtained by the correlation coefficient model and was relatively high, indicating good construct validity. The results of the construct validity of the SF-36 questionnaire are shown in Table 1. In addition, the coefficient range of the collective validity for all the scales was >0.4, except for the MH scale where the coefficient for the measured data was slightly low. Collective validity and divisional validity were considered to be good. The results of collective validity and divisional validity of each scale on the SF-36 questionnaire are shown in Table 2.

**Table 1:** Comparison of hypothetical and actual factor loadings.

	**Hypothetical factor loads**		**Actual factor loads**	
**Scales**	**PCS**	**MCS**	**PCS**	**MCS**
PF	+	–	0.736	0.273
RP	+	–	0.854	0.208
BP	+	–	0.891	0.247
GH	*	*	0.661	0.537
VT	*	*	0.692	0.485
SF	*	+	0.325	0.762
RE	–	+	0.247	0.800
MH	–	+	0.233	0.887

Correlation coefficient (*r*): *r* ≥ 0.70; * 0.70 > *r* > 0.30; ***–*** *r* ≤ 0.30

PCS, physical component summary; MCS, mental component summary; PF, physical function; RP, role-physical; BP, bodily pain; GH, general health; VT, vitality; SF, social function; RE, role-emotional ; MH, mental health.

**Table 2:** Collective validity and divisional validity of the SF-36 questionnaire.

		**Coefficient range**		**Collective validity**		**Divisional validity**	
**Scale**	**Number^*a*^**	**Collective validity**	**Divisional validity**	**Success/total ^*b*^**	**Success rate (%)**	**Success/total ^*c*^**	**Success rate (%)**
PF	10	0.531-0.906	0.375-0.936	10/10	100	80/80	100
RP	4	0.654-0.736	0.521-0.899	4/4	100	32/32	100
BP	2	0.915-0.933	0.439-0.971	2/2	100	16/16	100
GH	5	0.409-0.757	0.319-0.879	5/5	100	40/40	100
VT	4	0.410-0.612	0.274-0.812	4/4	100	32/32	100
SF	2	0.505-0.921	0.416-0.948	2/2	100	16/16	100
RE	3	0.754-0.804	0.508-0.894	3/3	100	24/24	100
MH	5	0.264-0.650	0.236-0.844	3/5	60	40/40	100

PF, physical function; RP, role-physical; BP, bodily pain; GH, general health; VT, vitality; SF, social function; RE, role-emotional ; MH, mental health.

a: Items per scale. b: Number of correlations between items and hypothesised scale corrected for overlap >0.40/total number of collective validity tests. c: Number of correlations significantly higher/total number of divisional validity tests.

*For collective validity, success means correlations between items and hypothesized scale corrected for overlap >0.40, and for divisional validity success means items in a dimension were more highly correlated with their hypothesized dimension than with other dimensions

### Reliability Analysis

The degree of internal uniformity among the items, namely the correlation between the items and the eight related scales, was expressed by Cronbach’s α coefficient (Table 3). The overall Cronbach’s α coefficient of the SF-36 questionnaire was 0.943, while the respective Cronbach’s α coefficients were all > 0.70. This met the requirement for group comparison. There was also a positive correlation between each of the eight scales of the SF-36 questionnaire (P < 0.01; Table 3). Table 3 shows the correlation coefficients (r) between the 36 items of the SF-36 questionnaire and the eight scales of study. 

**Table 3:** Internal uniform reliability and correlation coefficient of the SF-36 questionnaire.

	**Internal uniform reliability of SF-36 items with scales**		**Positive correlation between SF-36 scales**						
**Scale**	**Test-retest reliability (*n* = 40)**	**Cronbach’s α coefficient (*n* = 373)**	**PF**	**RP**	**BP**	**GH**	**VT**	**SF**	**RE**
PF	0.906^*^	0.951							
RP	0.913^*^	0.946	0.585^*^						
BP	0.869^*^	0.941	0.603^*^	0.604^*^					
GH	0.797^*^	0.899	0.442^*^	0.519^*^	0.635^*^				
VT	0.852^*^	0.817	0.416^*^	0.510^*^	0.614^*^	0.686^*^			
SF	0.744^*^	0.731	0.508^*^	0.593^*^	0.708^*^	0.583^*^	0.652^*^		
RE	0.902	0.952	0.441^*^	0.737^*^	0.478^*^	0.446^*^	0.483^*^	0.506^*^	
MH	0.751^*^	0.813	0.412^*^	0.501^*^	0.593^*^	0.644^*^	0.814^*^	0.664^*^	0.456^*^

^*^ *P* < 0.01. PF, physical function; RP, role-physical; BP, bodily pain; GH, general health; VT, vitality; SF, social function; RE, role-emotional ; MH, mental health.

The retest of the correlation between the items showed that r > 0.70 could be achieved for all eight scales (P < 0.01) (Table 4), demonstrating relatively good stability for the SF-36 questionnaire. The differences between the mean values for each scale after two rounds of measurements were not statistically significant. 

**Table 4:** Correlation coefficients between individual items and the eight scales of the SF-36 questionnaire.

**Dimensions**	**Correlation coefficient (r)**	***P*-value**
PF	0.646 - 0.912	< 0.01
RP	0.703 - 0.944	< 0.01
BP	0.725 - 0.871	< 0.01
GH	0.689 - 0.832	< 0.01
VT	0.796 - 0.865	< 0.01
SF	0.600 - 0.843	< 0.01
RE	0.625 - 0.913	< 0.01
MH	0.686 - 0.826	< 0.01

PF, physical function; RP, role-physical; BP, bodily pain; GH, general health; VT, vitality; SF, social function; RE, role-emotional ; MH, mental health.

 The split-half reliability measure was determined by splitting the SF-36 items in each dimension by an odd–even split, calculating the correlation coefficient r_1_ for each split separately, and comparing the two, thereby calculating the reliability of each part of the split questionnaire. This was corrected using the Spearman-Brown prediction formula r = 2r_1_/ (1 + r_1_), which generated the value of r = 0.784 (P < 0.001), showing that this questionnaire was relatively stable.

## Discussion

The SF-36 was originally developed as an instrument for health surveying which was widely used in studies of health-related QoL and medical outcomes surveys (MOS). Therefore, it is reasonable that the items in the SF-36 reflect more toward the scope of health statuses. However, the aim of WHOQOL-BREF was to capture a broad-ranging concept of QoL to the extent of incorporating environment domain in its scope[29]. There are also important differences between these two instruments. For example, items in the PF of the SF-36 cover a range of clearly specified mild to vigorous physical activities in comparison to a variety of aspects (e.g. pain, energy, sleep, mobility) in the physical domain of the WHOQOL-BREF. Some studies indicated that the SF-36 has more evidence supporting its use in HIV/AIDS than EQ-5D which would not be the best choice because of the pronounced ceiling effect[42]. 

There are only two studies on the quality of life of MSM population in China. Jun-rui Xia et al investigated the QoL of MSM in seven cities using the WHOQOL-BREF questionnaire[43]. The results showed that the reliability was acceptable except in the social relationships sub-scale. Yan-ming Sun et al assessed the QoL of MSM living with HIV/AIDS in Beijing city using the SF-36 questionnaire, which showed a good overall reliability, with all subscales exceeding 0.7[44]. Therefore, this study provided preliminary psychometric information for potential users of the SF-36 survey on Chinese MSM and for researchers who want to do further work. 

Our results obtained indicate that the SF-36 had acceptable reliability and validity in determining the QoL of Chinese MSM. The overall Cronbach’s α coefficient of the SF-36 questionnaire was 0.943, which indicated the Cronbach’s α values of SF-36 surveys were generally good and comparable to results of the general population and other populations from previous studies[25,45,46]. The Cronbach’s α values for internal consistency ranged from 0.731 to 0.952 across the scales of the SF-36 survey. Internal consistency reliability estimates for each scale exceeded 0.70 and supported the usefulness of the instruments in statistical analyses involving group comparison, which indicated a good internal uniformity. Some studies reported a lower Cronbach’s α coefficient (below 0.70) of SF scales among the general population[25-27,47]. Riley et al., found that all reliability coefficients exceeded 0.70 (range: 0.77-0.90) in HIV-infected homeless and marginally housed individuals[48]. Ping-Chuan Hsiung et al., found that the Cronbach’s a values for internal consistency ranged from 0.72 to 0.93 across the scales of the SF-36 in patients with HIV infection[29]. In our study, Cronbach’s α coefficient was above 0.70 for all the SF scales, which also indicated a good reliability in the determination of the QoL of the Chinese MSM population.

Our results indicated that the SF-36 questionnaire had applicable validity in the MSM population. The PCS and the MCS factor loads were in accordance with hypothetical correlations, which indicated good overall construct validity. Previous studies that were conducted among patients with HIV/AIDS showed good construct validities[29-31]. The scaling success rate on divisional validity was 100% for all scales, indicating definite scaling success. Our results showed that the assessment of collective validity was satisfactory in all scales except for the MH scale. The 9-4 and 9-8 item-scale correlations were just below the standard of 0.4 for item internal consistency in this study, which was not consistent with the outcomes of other surveys[29,49]. On one hand, this may be due to the stigma and discrimination against the MSM population in China and the fact that they may be reluctant to talk about their true feelings and emotions in public. On the other hand, the mental health domains did not have good correlations, possibly because of difficulties in interpreting the questions in the Chinese SF-36. Zhou B et al., also found the validity of MH in the 9-8 item was relative low for the Chinese elderly population[50]. Watkins et al, in developing a Vietnamese translation, had modified the conceptual definition of the MH scales to produce culturally more appropriate scales with clearer definitions[51]. For the MH scale, we suggest adding clear definitions of items relevant to the Chinese cultural context to improve subject understanding of the survey questions, uniformity in the responses, and the collective validity of the MH scale. 

However, we acknowledge that there are some limitations in this study. The participants were recruited from two cities of China, which may to some extent limit the representation of this study sample. Thus, it may have been more ideal to survey a larger sample size in order to better generalize the results from this study.

## Conclusion

The SF-36 has good reliability and acceptable validity in assessing the QoL of the MSM population in China, which could inform us of their health conditions, help us understand the related risk factors, and develop health policies to effectively prevent the spread of HIV in China. However, the collective validity of the mental health scale in items 9-4 and 9-8 was low. For future applications of this questionnaire, these two items of the mental health scale should be reconstructed with clearer definitions. 
